# High-Temperature Induced Sintering Strengthening of Mechanical Properties of Porous Silica: A Molecular Dynamics Study

**DOI:** 10.3390/gels12020125

**Published:** 2026-02-01

**Authors:** Ruoyu Bao, Yiming Song, Jiejie Shi, Yuanfu Zhang, Renhui Cheng, Mingyang Yang, Mu Du

**Affiliations:** 1 College of Safety Science and Engineering, Xi’an University of Science and Technology, Xi’an 710054, China; bry_2025@163.com (R.B.);; 2 School of Resources Engineering, Xi’an University of Architecture and Technology, Xi’an 710055, China; 3 Shenzhen Research Institute, Shandong University, Shenzhen 518057, China; 4 Institute for Advanced Technology, Shandong University, Jinan 250061, China

**Keywords:** silica aerogel, molecular dynamics, high temperature mechanical behavior, thermal runaway protection, pore evolution

## Abstract

Silica aerogels are critical for thermal protection in extreme environments; however, their mechanical response mechanisms under high temperatures remain elusive. This study employs large-scale molecular dynamics simulations to systematically investigate the mechanical behavior of silica aerogels (0.43–0.71 g/cm^3^) across a temperature range of 298–1800 K. The results reveal a fundamental competition between thermal softening and sintering-induced strengthening. Under tensile loading, the thermal softening effect dominates, leading to a significant fracture strength reduction of up to 49.6% at 1800 K, while simultaneously enhancing ductility, extending fracture strain to 80%. Conversely, under compressive loading, the sintering effect induced by temperatures above 900 K outweighs softening, resulting in a ~20% increase in the elastic modulus for high-density samples at 1300 K. Microstructural analysis attributes this enhancement to the preferential collapse of large pores and densification into an atomic-scale micropore range (0.5–1.0 nm). This work elucidates how the interplay between softening and sintering dictates material failure or strengthening, providing a microscopic theoretical basis for designing thermal shock-resistant materials for new energy batteries.

## 1. Introduction

Silica aerogels, characterized by a unique three-dimensional nanonetwork structure [1,2], are increasingly emerging as critical materials in frontier fields such as new energy safety [3], medical engineering [4], and ultra-high-temperature jet engine casings [5], owing to their ultra-low density, high porosity (>90%), and exceptional thermal and mechanical properties. Their distinctive physical performance is attributed to a specific nanoporous backbone architecture: a pearl-like network formed by interconnected amorphous silica particles. This structure not only constructs highly tortuous heat conduction paths, endowing the material with ultra-low thermal conductivity, but also provides an ultra-high specific surface area and abundant pore space [6]. Such microstructural features render silica aerogels an ideal barrier for blocking heat diffusion in thermal runaway protection for new energy batteries [7]. Simultaneously, their lightweight and porous nature offers a versatile structural foundation for designing intelligent flexible devices with shape memory functions and implantable biological scaffolds [8]. However, whether serving as shock protection during battery thermal runaway or enduring repeated deformation in flexible devices, the structural integrity of the material under complex loading conditions remains a prerequisite for its functionality [9]. Therefore, a profound understanding of the mechanical behavior of silica aerogels is not only fundamental to evaluating their service reliability but also key to realizing the transition from single-function thermal insulators to integrated load-bearing and thermal insulation materials.

The mechanical properties of silica aerogels determine the material’s resistance to elastic deformation, particularly under conditions of high stress concentration [10]. Currently, extensive research efforts have focused on the mechanical properties of aerogels, establishing a comprehensive landscape ranging from macroscopic experiments to microscopic simulations. Woignier et al. [11] established the classical power-law scaling relationship between aerogel density and elastic modulus, as well as fracture strength, through systematic three-point bending and compression tests, revealing the decisive influence of porosity on mechanical strength. Gross and Fricke [12] further confirmed the correlation between sound velocity and density using ultrasonic techniques and highlighted the non-linear elastic behavior of low-density aerogels under compression. As research advanced, Ma et al. [13] analyzed the contributions of backbone dangling bonds and ring structures to mechanical characteristics based on coarse-grained models. Furthermore, Patil et al. [14] utilized molecular dynamics simulations to not only validate the experimentally observed power-law relations but also to provide a deep atomic-scale analysis of the microscopic regulatory mechanisms involving nanopore size distribution, backbone connectivity, and Poisson’s ratio on the mechanical response. However, most of these studies have focused on the influence of single structural variables on mechanical properties at ambient temperature, neglecting the complex interplay of thermal–mechanical coupling effects in actual extreme service environments. Under high-temperature conditions, the aerogel backbone faces not only the risk of matrix thermal softening but also the potential for sintering neck growth between nanoparticles and local structural reorganization [15]. This dynamic microstructural evolution induced by high temperatures is bound to disrupt the mechanical scaling laws observed at room temperature. Therefore, there is an urgent need to conduct systematic molecular-scale investigations to unveil the specific mechanical evolution mechanisms and failure modes of aerogels under high temperatures.

Furthermore, despite the immense potential of silica aerogels in the aforementioned emerging fields, their intrinsic brittleness remains a fundamental bottleneck limiting their reliability in complex thermo-mechanical coupling environments [16,17,18]. In practical scenarios, such as battery thermal runaway or large deformation of flexible devices, materials often endure simultaneous severe thermal shock and mechanical loading [19]. Existing aerogel backbones struggle to maintain structural integrity under such extreme multi-physical field coupling, making them prone to catastrophic brittle fracture or structural collapse [20,21]. Although the incorporation of fibers or polymers can improve their room-temperature mechanical properties to a certain extent, these additives often introduce new interfacial thermal resistance or fail at high temperatures, thereby increasing the complexity and uncertainty of microstructural design [22]. Addressing this challenge, the academic community has conducted preliminary explorations into the correlation between heat and mechanics. Woignier et al. [23] confirmed that heat treatment can significantly alter the topological structure of aerogels and enhance their room-temperature mechanical modulus by inducing pore collapse and density increase through high-temperature sintering pretreatment. Wang et al. [24], through in situ thermogravimetry and microscopic characterization, revealed that high temperatures (>1000 °C) cause severe mass loss and the derivation of microscopic defects in the nanobackbone, thereby affecting structural integrity. However, existing studies mostly focus on residual properties after heat treatment or thermal stability without mechanical loading, failing to capture the dynamic response of the material in real-time during high-temperature loading. At the instant of real battery thermal runaway, the interior of the aerogel backbone is governed by the complex coupling of two mechanisms: thermal softening and thermal sintering [25]. Macro-experiments, however, struggle to distinguish the dominating temperature ranges and critical evolution points of these two mechanisms, resulting in a lack of clear theoretical guidance for microstructural design under specific extreme operating conditions. Therefore, it is necessary to conduct atomic-scale molecular dynamics simulations to elucidate from a microscopic mechanism how thermal and force fields synergistically lead to material failure or strengthening, thus filling this theoretical gap.

MD simulations, with their high resolution of atomic trajectories, have become a powerful tool for overcoming the limitations of experimental observation and analyzing the micromechanical behavior of aerogels. For instance, Kieffer and Angel [26] pioneered atomic-scale research by constructing a non-fractal silica network model using the negative pressure rupturing method. Subsequently, Campbell et al. [27], based on more accurate atomic potential functions, established the classical power-law scaling relationship between aerogel density and elastic modulus, laying the foundation for theoretical prediction. In the era of large-scale computing, Gelb et al. [28] further utilized million-atom systems to deeply investigate the microscopic regulatory mechanisms of nanopore size distribution on material stiffness and fracture behavior. In particular, Patil et al. [29] successfully reproduced the experimentally observed tensile brittleness and compressive densification behavior using a melt-quench-expansion modeling strategy and quantified the pore wall deformation mechanisms at different densities. However, existing MD studies mostly focus on the low-temperature elastic region or small deformation stages, with few works systematically revealing the topological evolution laws of the aerogel nanobackbone under tensile and compressive loads across the range from room temperature to near the melting point (>1500 K). Specifically, there is currently a lack of systematic understanding regarding how the two competitive mechanisms of thermal softening and structural sintering under high temperatures jointly determine the macroscopic mechanics of the material. This lack of knowledge regarding the mechanical response mechanism across the full temperature range leaves microstructural design for specific extreme conditions, such as the moment of battery thermal runaway, in a predicament [30,31].

Despite significant advancements in characterizing the ambient mechanical properties of silica aerogels, a critical knowledge gap persists regarding their real-time thermo-mechanical response under extreme thermal environments. The existing literature predominantly focuses on either room-temperature scaling laws or the residual mechanical properties of samples after heat treatment, thereby failing to capture the dynamic interplay of atomic-scale processes during high-temperature loading. Table 1 provides a comparative summary of prior experimental and computational studies, further highlighting the lack of integrated thermo-mechanical investigations. Specifically, there is a notable absence of systematic molecular dynamics (MD) studies that concurrently capture both tensile and compressive behaviors across a wide temperature spectrum, particularly at near-melting temperatures (up to 1800 K). Furthermore, the detailed analysis of real-time pore evolution and its correlation with loading-dependent failure modes remain unexplored in current coupled simulations. The inability to distinguish the dominating regimes between the thermal softening of the silica matrix and the high-temperature sintering of the backbone hinders the systematic design of aerogel structures for critical safety applications, such as thermal runaway protection. Consequently, there is an urgent need to elucidate how thermal and mechanical fields synergistically dictate material failure or strengthening through atomistic investigations.

In this study, large-scale molecular dynamics simulations of approximately 192,000 atoms were performed on silica aerogels of various densities to investigate their tensile and compressive behaviors from 298 K to 1800 K. Beyond quantifying the non-linear regulation of temperature and density on mechanical strength and dynamically reconstructing pore evolutionary trajectories, this work systematically identifies the real-time competitive mechanism between thermal softening and sintering-induced strengthening. This investigation reveals how loading modes uniquely dictate thermo-mechanical responses, providing a microscopic theoretical framework that correlates atomistic-level structural reorganizations with the critical failure stages of battery systems. Ultimately, this research establishes a robust foundation for designing next-generation thermal safety materials tailored for extreme environments.

## 2. Results and Discussion

### 2.1. Tensile Fracture Behavior and Stress Response

**Figure 1:**
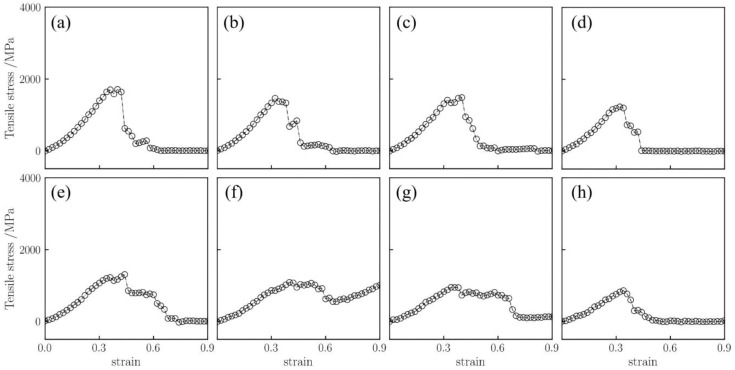
Uniaxial tensile stress–strain curves for silica aerogel with a density of 0.43 g/cm^3^ at temperatures of (**a**) 298 K, (**b**) 450 K, (**c**) 600 K, (**d**) 750 K, (**e**) 900 K, (**f**) 1300 K, (**g**) 1500 K, and (**h**) 1800 K.

The fracture strength of silica aerogel models under uniaxial tension was investigated. Figure 1 displays the tensile stress–strain curves for the silica aerogel with a density of 0.43 g/cm^3^ at different temperatures (298–1800 K).

**Figure 2:**
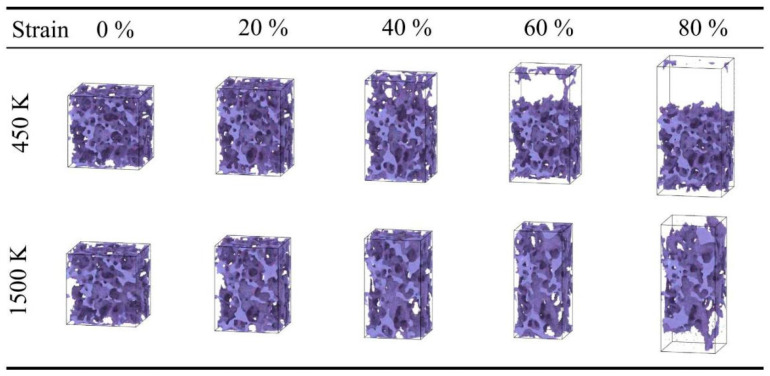
Snapshots of silica aerogel with a density of 0.43 g/cm^3^ under uniaxial tension at 450 and 1500 K.

For the low-density silica aerogel (0.43 g/cm^3^) (Figure 1), three distinct deformation stages are observed within the range of 298 K to 1800 K: a linear elastic stage, a non-linear plastic deformation stage, and a fracture stage [37]. At 298 K (Figure 1a), the stress increases linearly with strain in the initial stage (0–5%). When the strain exceeds 5%, the slope of the curve gradually decreases, entering a prolonged non-linear deformation stage. The stress reaches its peak at approximately 45% strain, after which the material enters the fracture stage, culminating in complete rupture at around 60% strain. As the temperature rises, the fracture process of the material is prolonged. At a high temperature of 1500 K (Figure 1g), the stress decline after fracture becomes more gradual, and the strain at complete rupture increases to approximately 70%. The atomic snapshots in Figure 2 visually illustrate this change. It is important to note that these snapshots are rendered directly from the real-time atomic coordinates recorded in the MD simulation trajectories. By visualizing the instantaneous positions of all atoms frame-by-frame, Figure 2 faithfully reproduces the actual topological evolution of the aerogel backbone, providing direct microscopic evidence that corroborates the macroscopic mechanical transition analyzed above. At 450 K with 40% strain, the aerogel backbone is on the verge of fracture, with atomic clusters beginning to separate; whereas at 1500 K, the atomic backbone near the fracture region is significantly elongated, forming slender filamentary structures at the connecting points prior to rupture. This microscopic elongation phenomenon aligns with the macroscopic trend of a prolonged fracture process and gradual stress decline at high temperatures.

Furthermore, increasing temperature leads to a gradual reduction in the maximum stress of the silica aerogel. As shown in Figure 1, the maximum stress decreases progressively from 1704.2 MPa as the temperature rises from 298 K. When the temperature reaches 1500 K, the maximum stress drops to 947.1 MPa, and the strain at peak stress advances to approximately 40%. At the high temperature of 1800 K (Figure 1h), the maximum stress further decreases to approximately 859.5 MPa. Compared to 298 K, the maximum stress at 1800 K is reduced by approximately 844.7 MPa, representing a decrease of 49.6%.

**Figure 3:**
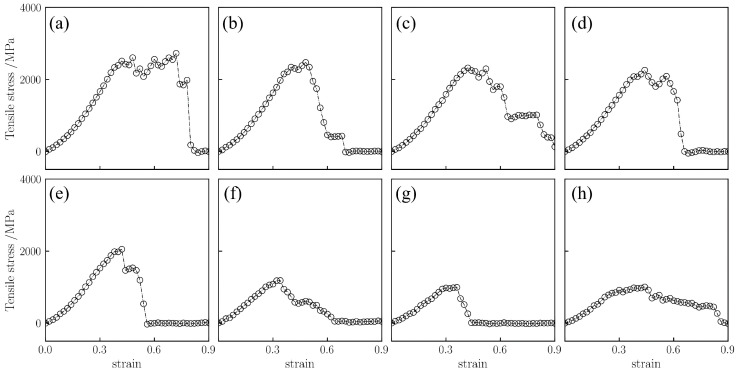
Uniaxial tensile stress–strain curves for silica aerogel with a density of 0.50 g/cm^3^ at temperatures of (**a**) 298 K, (**b**) 450 K, (**c**) 600 K, (**d**) 750 K, (**e**) 900 K, (**f**) 1300 K, (**g**) 1500 K, and (**h**) 1800 K.

As shown in Figure 3, the medium-density silica aerogel (0.50 g/cm^3^) exhibits variation trends similar to those of the low-density sample (0.43 g/cm^3^). However, due to the increased density, the slope of the curve in the initial stage (strain 0–5%) is steeper. Additionally, high temperature similarly delays the fracture process. As shown in Figure 3h, at 1800 K, the stress decline after fracture tends to be gentle, and the strain at complete rupture is postponed to approximately 80%. In terms of load-bearing capacity, this density sample exhibits higher stress. As shown in Figure 3a, at 298 K, its peak stress reaches 2511.4 MPa. With increasing temperature, the peak stress begins to decrease, falling to 1000.4 MPa at 1500 K and maintaining approximately 1008.6 MPa at 1800 K. Compared to 298 K, the peak stress at 1800 K decreases by 59.8%. This attenuation magnitude is significantly higher than the 49.6% observed for the low-density sample (0.43 g/cm^3^), indicating that the thermal stability of the material’s strength diminishes as density increases.

**Figure 4:**
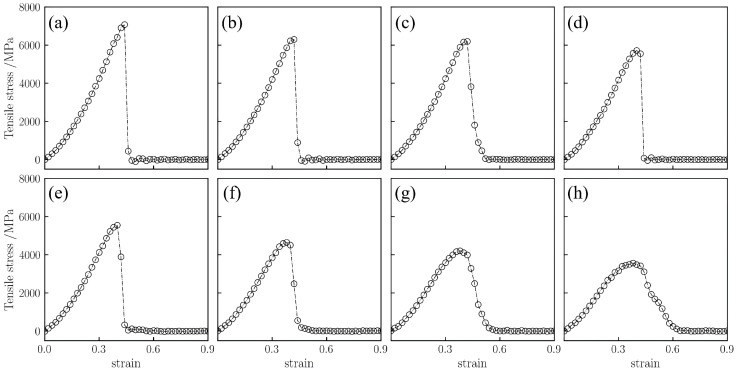
Uniaxial tensile stress–strain curves for silica aerogel with a density of 0.71 g/cm^3^ at temperatures of (**a**) 298 K, (**b**) 450 K, (**c**) 600 K, (**d**) 750 K, (**e**) 900 K, (**f**) 1300 K, (**g**) 1500 K, and (**h**) 1800 K.

**Figure 5:**
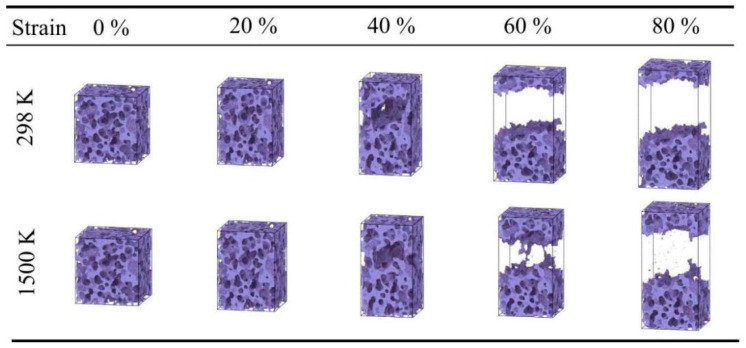
Snapshots of silica aerogel with a density of 0.71 g/cm^3^ under uniaxial tension at 298 K and 1500 K.

As shown in Figure 4, the high-density silica aerogel (0.71 g/cm^3^) displays more pronounced brittle characteristics. Compared to the low- and medium-density samples, the stress–strain curve of the high-density aerogel drops more rapidly after reaching the peak. As shown in Figure 4a, at 298 K, the material rapidly reaches peak stress after a short period of elastic deformation, followed by a vertical drop in stress to zero, indicating a very brief fracture process. Even at a high temperature of 1800 K (Figure 4h), although the strain at fracture increases slightly, the stress decline remains rapid. The atomic snapshots in Figure 5 show that, unlike the filamentary connections observed in the low-density sample at 60% strain, the high-density sample exhibits bulk separation at fracture. Whether at room temperature or high temperature, at the moment of fracture, the backbone structure undergoes large-scale separation with relatively flat fracture surfaces, and no obvious atomic cluster elongation is observed. This microscopic rapid separation corresponds directly to the steep drop in stress after the peak on the macroscopic curve.

In terms of load-bearing capacity, the high-density sample (0.71 g/cm^3^) exhibits a higher fracture strength. As shown in Figure 4, at 298 K, its peak stress reaches as high as 7080.6 MPa. However, as the temperature rises, its strength decreases significantly. By 1500 K, the peak stress drops to 4210.1 MPa, and further falls to 3566.2 MPa at 1800 K. Compared to 298 K, the peak stress at 1800 K decreases by approximately 49.6%.

### 2.2. Compressive Densification and High-Temperature Strengthening

**Figure 6:**
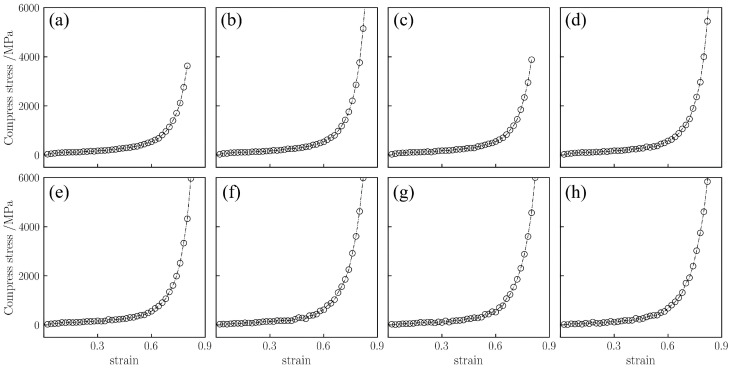
Uniaxial compressive stress–strain curves for silica aerogel with a density of 0.43 g/cm^3^ at temperatures of (**a**) 298 K, (**b**) 450 K, (**c**) 600 K, (**d**) 750 K, (**e**) 900 K, (**f**) 1300 K, (**g**) 1500 K, and (**h**) 1800 K.

Unlike the fracture observed under tension, silica aerogels exhibit different behaviors under uniaxial compression. Figure 6 displays the compressive stress–strain curves for the silica aerogel model with a density of 0.43 g/cm^3^ at 298–1800 K. Regardless of density or temperature, the compression process of silica aerogels can be divided into three stages [34]: (1) the linear elastic stage, where the backbone undergoes elastic deformation; (2) the yielding stage, where pores collapse and stress increases slowly with strain; and (3) the densification stage, where the backbone is compacted and stress rises rapidly. This indicates that even at high temperatures, the aerogel backbone does not completely disintegrate but maintains load-bearing capacity through pore closure.

As shown in Figure 6, the low-density silica aerogel (0.43 g/cm^3^) exhibits these three stages within the range of 298 K to 1800 K. Taking 298 K as an example (Figure 6a), in the initial loading phase (strain 0–10%), stress increases linearly with strain. When strain exceeds 10%, the curve enters a long yielding stage lasting until approximately 65% strain, during which the stress increase is very slow, indicating extensive collapse of the pore structure. After the strain exceeds 65%, the curve enters the densification stage, and stress rises rapidly. When the strain reaches 80%, the compressive stress is approximately 3630.8 MPa.

**Figure 7:**
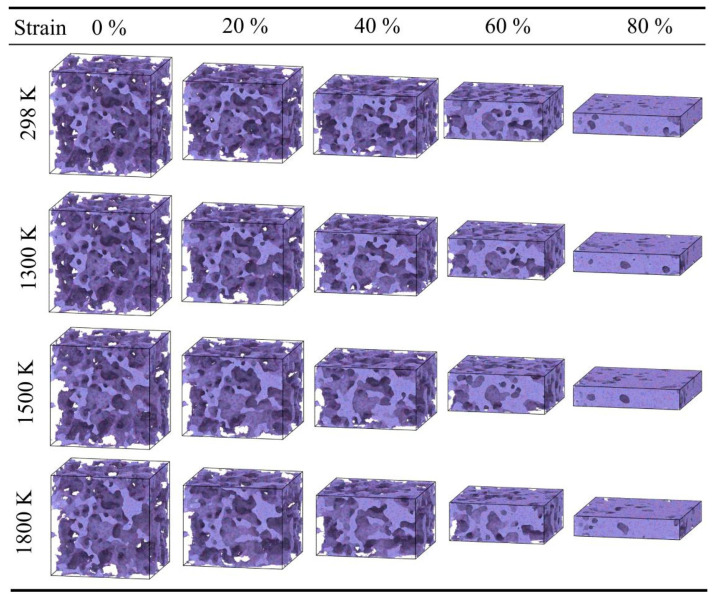
Snapshots of silica aerogel with a density of 0.43 g/cm^3^ under uniaxial compression at 298 K, 1300 K, 1500 K, and 1800 K.

Furthermore, temperature has a significant influence on the stress in the densification stage. Within the range of 298 K to 900 K, the mechanical behavior of the material is relatively stable, with the stress at 80% strain maintaining between 3500 MPa and 3800 MPa. However, when the temperature rises to 1500 K (Figure 6g), the rate of stress increase in the densification stage accelerates, and the stress at 80% strain rises to approximately 4600.8 MPa. At 1800 K (Figure 6h), the stress at 80% strain is approximately 4614.28 MPa. The atomic snapshots in Figure 7 illustrate the morphological differences at different temperatures. At 298 K, the compression process is mainly characterized by pore collapse and particle stacking, with clear particle boundaries. In contrast, at high temperatures of 1300–1800 K, the backbone structure shows obvious fusion; the originally independent particle boundaries become blurred and interconnected, and the number of small pores decreases, forming a more continuous bulk. This microscopic fusion feature corresponds to the phenomenon of increased stress at high temperatures in the macroscopic curves.

Atomic-scale bond breaking and re-forming observed at high temperatures reflect the intrinsic microscopic mechanisms of silica sintering. From a thermodynamic standpoint, sintering is driven by the reduction in the system’s total surface free energy. For amorphous silica, this energy optimization is facilitated by localized Si-O bond rearrangements via atomic diffusion. Molecular dynamics studies demonstrate that these structural adjustments under thermal influence can increase the final density of porous silica by 30%, showing the influence of microscopic bond evolution on macroscopic characteristics. Although the molecular dynamics timescale is limited to nanoseconds and remains shorter than experimental sintering durations, the captured bond-switching events effectively characterize the early stages of sintering neck formation and the structural densification process. Therefore, the bond dynamics reported here provide a mechanistic foundation for the thermally induced strengthening of the aerogel backbone.

**Figure 8:**
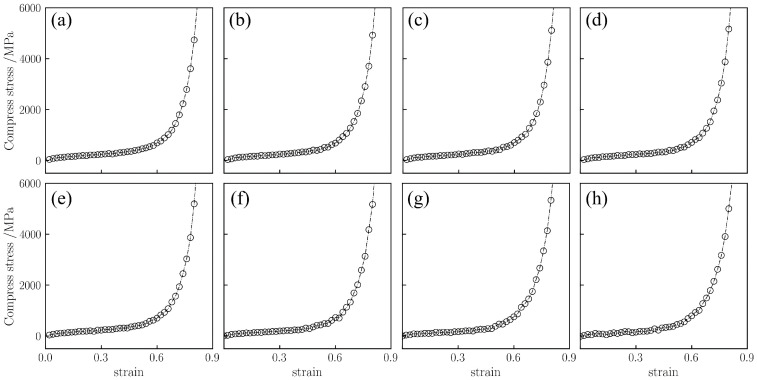
Uniaxial compressive stress–strain curves for silica aerogel with a density of 0.50 g/cm^3^ at temperatures of (**a**) 298 K, (**b**) 450 K, (**c**) 600 K, (**d**) 750 K, (**e**) 900 K, (**f**) 1300 K, (**g**) 1500 K, and (**h**) 1800 K.

As shown in Figure 8, the medium-density silica aerogel (0.50 g/cm^3^) exhibits similar three stages, but the increased density leads to an earlier onset of the densification stage. Comparing Figure 6 (0.43 g/cm^3^), the yielding stage of the low-density sample lasts until 65% strain, whereas the medium-density sample ends yielding and enters the densification stage at approximately 60% strain. In terms of stress, the medium-density sample has a higher load-bearing capacity. As shown in Figure 8a, at 298 K with 80% strain, the stress is 4738 MPa. At 1800 K (Figure 8h), the stress rises faster, increasing to 5300.4 MPa at 80% strain, which is significantly higher than the corresponding value for the low-density sample at the same temperature (4614.3 MPa).

**Figure 9:**
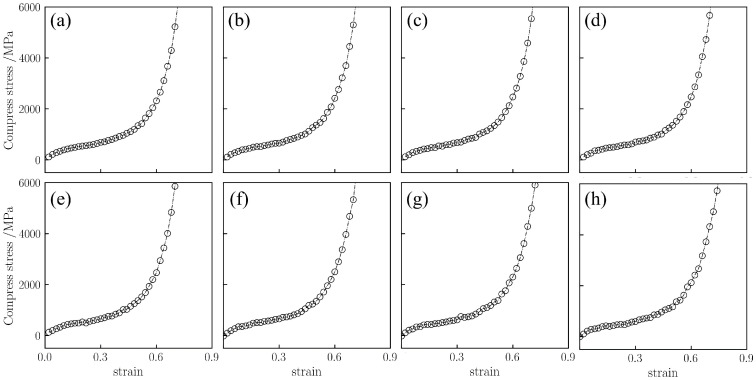
Uniaxial compressive stress–strain curves for silica aerogel with a density of 0.71 g/cm^3^ at temperatures of (**a**) 298 K, (**b**) 450 K, (**c**) 600 K, (**d**) 750 K, (**e**) 900 K, (**f**) 1300 K, (**g**) 1500 K, and (**h**) 1800 K.

**Figure 10:**
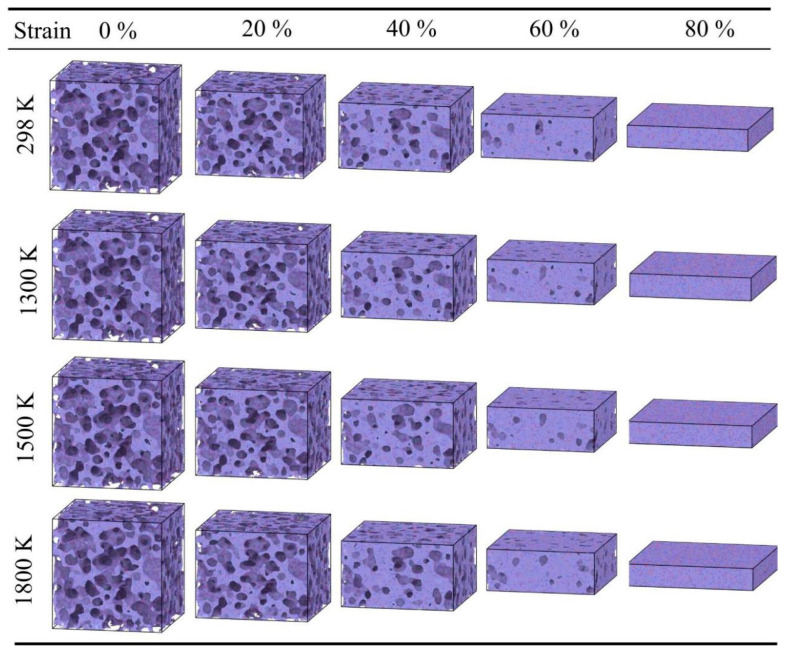
Snapshots of silica aerogel with a density of 0.71 g/cm^3^ under uniaxial compression at 298 K, 1300 K, 1500 K, and 1800 K.

As shown in Figure 9, the densification stage of the high-density silica aerogel (0.71 g/cm^3^) advances further. This sample has a shorter yielding stage, starting densification at approximately 50% strain. This indicates that after brief compression, the internal pores rapidly disappear, and the backbone enters a solid compression state. Furthermore, as shown in Figure 9a, when the strain reaches 70%, the compressive stress at 298 K is 5226.9 MPa, significantly higher than that of the low-density sample. As shown in Figure 10, comparing the atomic configuration at 0% strain, the backbone of the high-density sample is denser, with large pores being almost invisible. At 80% strain, the high-density sample has been essentially compacted into a dense solid, with pores almost completely vanishing; in contrast, other density samples still retain some pores at this strain. This state of thorough compaction enables the high-density material to exhibit higher load-bearing capacity in the later stages of compression.

### 2.3. Porosity Evolution

**Figure 11:**
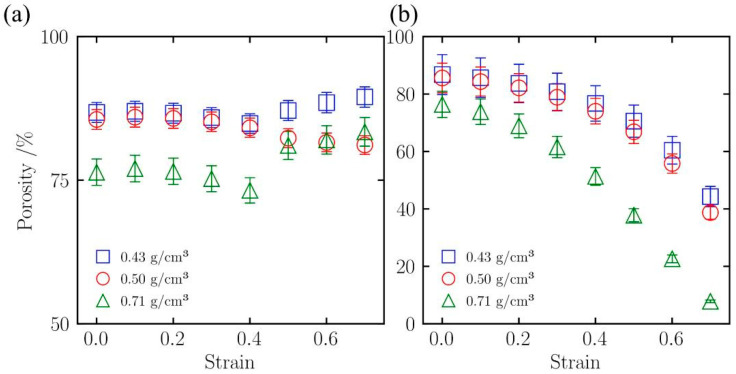
Evolution of porosity for silica aerogels with different densities under (**a**) uniaxial tension and (**b**) uniaxial compression.

The change in porosity reflects the structural alteration of the aerogel backbone under loading [38]. Figure 11 displays the variation in porosity with strain for silica aerogels of different densities (0.43, 0.50, and 0.71 g/cm^3^) under uniaxial tension (Figure 11a) and compression (Figure 11b).

Figure 11a shows the porosity change under uniaxial tension. The variation in porosity during tension is small, but differences in density lead to divergent trends. For the low-density sample (0.43 g/cm^3^), porosity decreases slowly with increasing strain, dropping gradually from an initial value of approximately 82%, indicating that the backbone maintains connectivity during tension. However, with increasing density, this trend changes. In the medium-density sample (0.50 g/cm^3^), porosity stops decreasing and begins to rise slightly when the strain exceeds 40%. This upward trend is more pronounced in the high-density sample (0.71 g/cm^3^), where the curve shows a clear inflection point in the late stage of tension (strain > 40%) and porosity increases significantly, suggesting that volume expansion or internal cavity enlargement occurs in medium- and high-density samples under tension.

In contrast, Figure 11b shows a continuous decrease in porosity under compression. The change in porosity corresponds to the stress–strain curve, similarly exhibiting three stages: linear elastic, yielding, and densification. Increased density leads to an earlier densification stage and a shorter yielding stage. As shown in Figure 11b, for the low-density sample (0.43 g/cm^3^), the yielding stage is longer, persisting until approximately 65% strain before entering the densification stage, and it still retains approximately 15% porosity at 80% strain. Conversely, the high-density sample (0.71 g/cm^3^) has a very short yielding stage, and porosity decreases more rapidly, showing a clear turning point at approximately 50% strain. As compression continues to near 70% strain, its porosity drops to below 5%, indicating that the material has largely completed the densification process at this strain.

**Figure 12:**
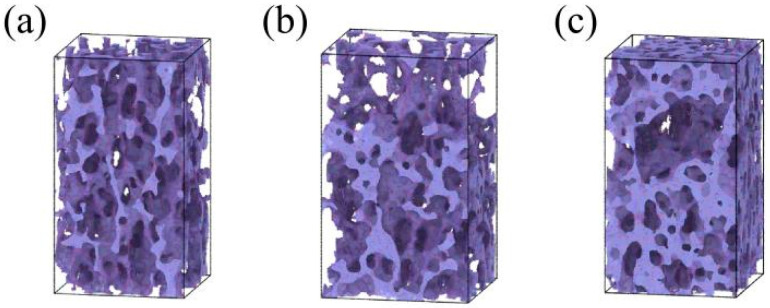
Snapshots of silica aerogel models with densities of (**a**) 0.43 g/cm^3^, (**b**) 0.50 g/cm^3^, and (**c**) 0.71 g/cm^3^ at 40% strain.

Figure 12 displays snapshots of the internal backbone of silica aerogels with different densities at 40% tensile strain at room temperature (298 K). As density increases, the structure exhibits different characteristics. As shown in Figure 12a, for the low-density sample (0.43 g/cm^3^), the backbone elongates significantly in the tensile direction, but the overall structure remains continuous without obvious fracture or large cracks, consistent with the continuous decrease in porosity data. In contrast, the medium-density sample (0.50 g/cm^3^) (Figure 12b) begins to show structural damage, with enlarged pores appearing locally in the backbone. In the high-density sample (0.71 g/cm^3^) (Figure 12c), this damage evolves into visible failure; the snapshot clearly shows the formation of large voids and cracks inside the material, and the backbone has undergone severe separation.

**Figure 13:**
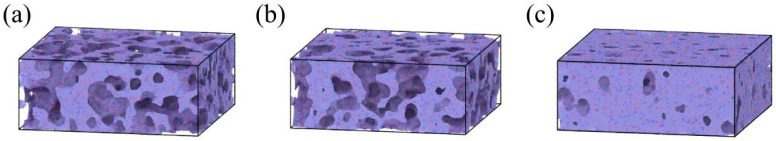
Snapshots of silica aerogel models with densities of (**a**) 0.43 g/cm^3^, (**b**) 0.50 g/cm^3^, and (**c**) 0.71 g/cm^3^ at 60% strain.

Figure 13 displays snapshots of the internal backbone of silica aerogels with different densities at 60% compressive strain at room temperature (298 K). This figure illustrates the differences in the degree of densification among samples of different densities. As shown in Figure 13a, for the low-density sample (0.43 g/cm^3^), although the backbone is significantly compressed, numerous unclosed pores can still be clearly observed internally. This state indicates that the material is still in the late stage of yielding and has not yet fully densified, which aligns with the porosity data at this strain in Figure 11b. Conversely, for the high-density sample (0.71 g/cm^3^) (Figure 13c), at the same strain, the original pore structure has almost completely disappeared, and the backbone is squeezed into a continuous and compact solid with very tight atomic packing. This indicates that the high-density sample has largely completed densification at this strain, consistent with the changes in the porosity curve in Figure 11b.

It is worth noting that the applied strain rate of 10^9^ s^−1^ in this MD study differs from typical experimental conditions, a factor that influences the absolute magnitude of the mechanical properties. Physically, the rapid loading timescale limits long-term relaxation mechanisms, such as viscous flow, which typically accommodate stress in quasi-static experiments. Consequently, the absolute strength values reported herein represent the material’s instantaneous stiffness and likely constitute theoretical upper bounds compared to experimental measurements. Conversely, the fracture strain and high-temperature ductility might be conservatively underestimated, as the limited simulation window restricts diffusion-mediated elongation that would otherwise occur over longer timescales. However, despite these shifts in absolute values, the observed trends regarding high-temperature sintering strengthening remain robust. As the high strain rate imposes a strict condition on diffusion-controlled sintering, the observed increase in elastic modulus implies that the actual strengthening effect in real-world scenarios could be more pronounced than predicted here.

### 2.4. Elastic Modulus

The elastic modulus, as a fundamental physical quantity measuring material stiffness and resistance to deformation, directly reflects the structural stability of the silica aerogel backbone under thermo-mechanical coupling fields [39]. To deeply investigate the regulatory mechanisms of density and temperature on the mechanical properties of aerogels, this section provides statistics on the elastic response of models with different densities (0.43, 0.50, and 0.71 g/cm^3^) within the range of 298 to 1800 K. All elastic modulus values were calculated by extracting the slope of the initial linear elastic stage of the stress–strain curves.

#### 2.4.1. Model Verification

**Figure 14:**
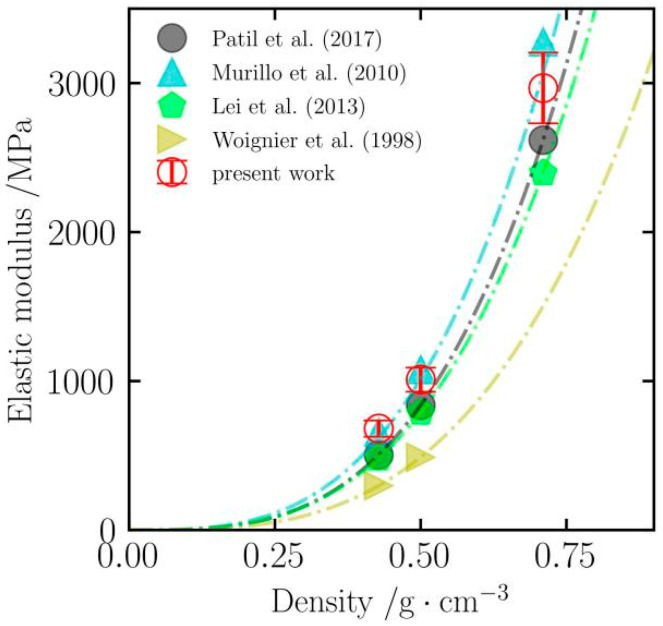
Comparative verification of the elastic modulus of silica aerogels with different densities at 300 K. The figure shows the comparison between the simulation results of this study and existing molecular dynamics simulation data (Patil et al. [34], Murillo et al. [40], Lei et al. [35]) and experimental measurement data (Woignier et al. [41]).

To verify the accuracy of the silica aerogel model constructed in this paper, the elastic moduli simulated at 300 K were compared with existing simulation studies (Patil et al., Murillo et al., Lei et al.). Figure 14 displays the distribution of elastic moduli at different densities. As shown, the elastic modulus calculated in this study increases significantly with increasing density, and the values show a high degree of agreement with existing simulation data. At low density (0.43 g/cm^3^), the elastic modulus in this study is 681.7 MPa, which is very close to the 658.9 MPa reported by Murillo et al. [40], with a deviation of only 3.5%. As the density increases to 0.71 g/cm^3^, the elastic modulus value in this study is 2968.4 MPa. This value falls between the results of Lei et al. [35] and Murillo et al. [40], with deviations of 23.8%, 13.3% (relative to Patil et al. [34]), and 9.4% from Lei et al., Patil et al., and Murillo et al., respectively, all of which are within a reasonable range of simulation error.

Furthermore, the simulation results of this study were compared with the experimental data of Woignier et al. [41]. At densities of 0.43 and 0.50 g/cm^3^, the calculated values in this study are all higher than the experimental measurements. This discrepancy is expected in MD simulations; the main reason is that the atomic models constructed in simulations are generated based on idealized structures with relatively perfect internal structures, whereas real aerogel materials often contain macroscopic cracks or structural defects, which significantly reduce the actual mechanical strength of the material. Although there are numerical differences, the trend of strengthening with increasing density is consistent for both, indicating that the model can reflect the basic mechanical characteristics of silica aerogels.

#### 2.4.2. Thermal Softening Effect Under Tension

**Figure 15:**
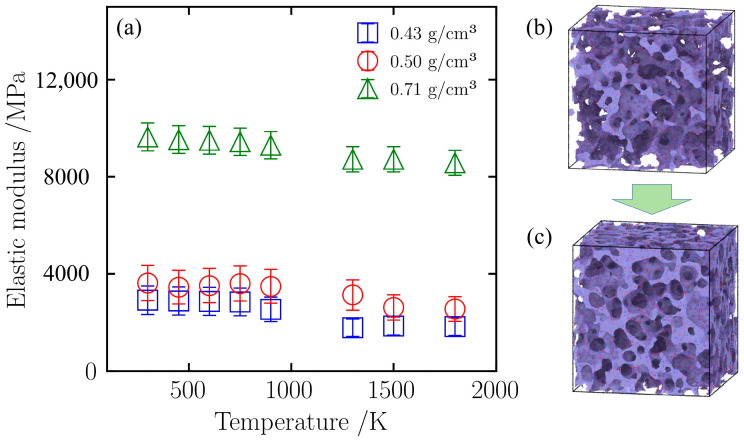
(**a**) Elastic modulus of silica aerogels with different densities under uniaxial tension; (**b**) Snapshot of the initial model with a density of 0.43 g/cm^3^; (**c**) Snapshot of the initial model with a density of 0.71 g/cm^3^.

Density significantly affects the elastic modulus of silica aerogels. As shown in Figure 15a, at room temperature (298 K), the elastic modulus increases with density. The elastic modulus of the low-density (0.43 g/cm^3^) sample is 2919.1 MPa, while that of the high-density (0.71 g/cm^3^) sample reaches 9641.1 MPa, an increase of more than threefold. Combining this with the initial atomic configurations in Figure 15b,c, it can be seen that the backbone of the low-density sample is relatively sparse with many unconnected regions; in contrast, the atomic packing of the high-density sample is tighter, forming a backbone network with better connectivity. This difference in structural compactness directly leads to the higher elastic modulus exhibited by the high-density sample in the initial tensile stage.

Despite the high-density sample having a higher initial elastic modulus, the tensile elastic moduli of all samples show a downward trend with increasing temperature, which is mainly attributed to the thermal softening effect [42]. As shown in Figure 15a, regardless of density, the tensile elastic modulus of all samples decreases with increasing temperature. Taking the 0.71 g/cm^3^ sample as an example, although its absolute stiffness is high, during the heating process from 298 K to 1800 K, its elastic modulus falls from 9641.1 MPa to 8574.9 MPa, a cumulative decrease of approximately 11.1%. This process does not proceed linearly at a constant rate; within the medium-to-high temperature range of 900 K to 1300 K, the material exhibits a more severe decay in elastic modulus. Quantitative analysis shows that the decay rate of the elastic modulus in this interval is 1.44 MPa/K, which is significantly higher than the decay rates in the low-temperature stage (0.57 MPa/K) and the high-temperature stage (0.30 MPa/K). This phenomenon indicates that within this temperature range, topological relaxation and weak bond dissociation may occur in the microscopic network of the silica aerogel. The intense atomic thermal vibration excited by high temperatures weakens the Si-O bond energy, triggering the fracture of some metastable connection structures. This concentrated bond weakening effect dominates the decline in macroscopic elastic modulus [43]. Therefore, even a backbone network reinforced by densification cannot completely offset the material’s elastic modulus degradation caused by high temperatures.

#### 2.4.3. Sintering Strengthening Effect Under Compression

**Figure 16:**
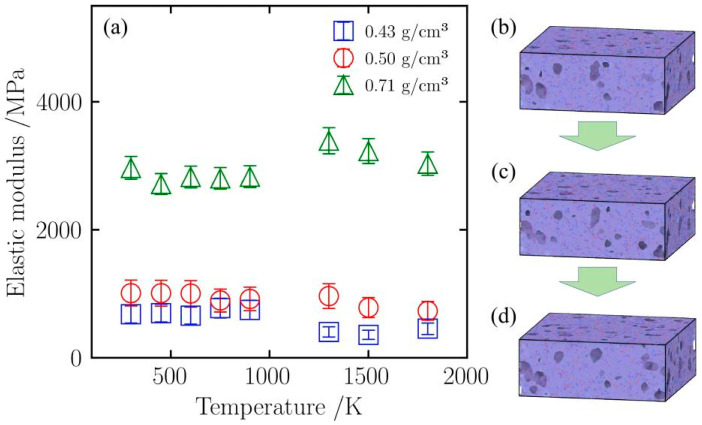
(**a**) Elastic modulus of silica aerogels with different densities under uniaxial compression; (**b**) Snapshot of the initial model with a density of 0.43 g/cm^3^; (**c**) Snapshot of the initial model with a density of 0.50 g/cm^3^; (**d**) Snapshot of the initial model with a density of 0.71 g/cm^3^.

Unlike the trend of elastic modulus change under tensile loading, the elastic modulus of silica aerogels shows an upward trend under high-temperature compression. As shown in Figure 16a, in the medium-to-low temperature range of 298 K to 900 K, the compressive elastic moduli of samples of all densities remain relatively stable with small fluctuations, indicating that the backbone structure has not yet undergone major changes in this temperature range. However, when the temperature exceeds 900 K, samples of different densities exhibit different mechanical responses. For the low-density (0.43 g/cm^3^) sample, its elastic modulus begins to decrease in the 900 K to 1300 K range, dropping from 748.6 MPa to 404.2 MPa, a decrease of 46%. This attenuation indicates that its sparse backbone network cannot withstand the matrix thermal softening induced by high temperatures, leading to a reduction in elastic modulus [32]. In contrast, with increasing density, the elastic modulus of the medium-density (0.50 g/cm^3^) sample stops decreasing and rebounds in this interval, rising from 920.0 MPa at 900 K to 964.4 MPa at 1300 K, indicating that the sintering phenomenon has begun to intervene and enhance the material’s intrinsic strength [33]. This trend is more pronounced in the high-density (0.71 g/cm^3^) sample, where the material’s elastic modulus jumps from 2831.3 MPa at 900 K to 3392.1 MPa at 1300 K, an increase of approximately 20%, and at this point, the material’s elastic modulus surpasses that at room temperature (2968.4 MPa). This strengthening effect, which becomes more significant with increasing density, indicates that silica aerogels may undergo a sintering reaction at 900 K, which enhances the material’s intrinsic strength, causing the rise in elastic modulus.

The atomic snapshots in Figure 16b–d illustrate this process. Comparing the structures at 900 K (Figure 16b) and 1300 K (Figure 16c) under the same compressive strain (60%) reveals that at 900 K, although the backbone is compressed, there are still relatively distinct boundaries between particles. At this point, sintering has not yet occurred significantly, and thermal softening leads to a lower elastic modulus. In contrast, in the snapshots at 1300 K (Figure 16c) and 1800 K (Figure 16d), the backbone structure shows fusion; the originally independent particle boundaries become blurred and interconnected, and pores become fewer. This phenomenon indicates that high temperatures lead to sintering, enhancing the continuity of the backbone. It is precisely this structural enhancement brought about by sintering that causes the material’s elastic modulus to peak at 1300 K. Even at 1800 K (Figure 16d), although higher temperatures exacerbate thermal softening, thanks to the dense structure formed by sintering, its elastic modulus remains at a high level of 3034.3 MPa.

### 2.5. Pore Size Distribution

The macroscopic mechanical behavior of silica aerogels has been analyzed above; this section will further elucidate the microstructural mechanisms behind tensile fracture and compressive strengthening through statistical results of pore size distribution. Pore size is defined as the diameter of the largest sphere that can contain a given point without overlapping with adjacent wall atoms [34]. To clearly discern the specific contributions of pores at different scales during deformation, this section divides the pore structure into three parts: small pores (<1.5 nm), mesopores (1.5–3.0 nm), and large pores (>3.0 nm) [36].

#### 2.5.1. Pore Evolution Under Tensile Loading

**Figure 17:**
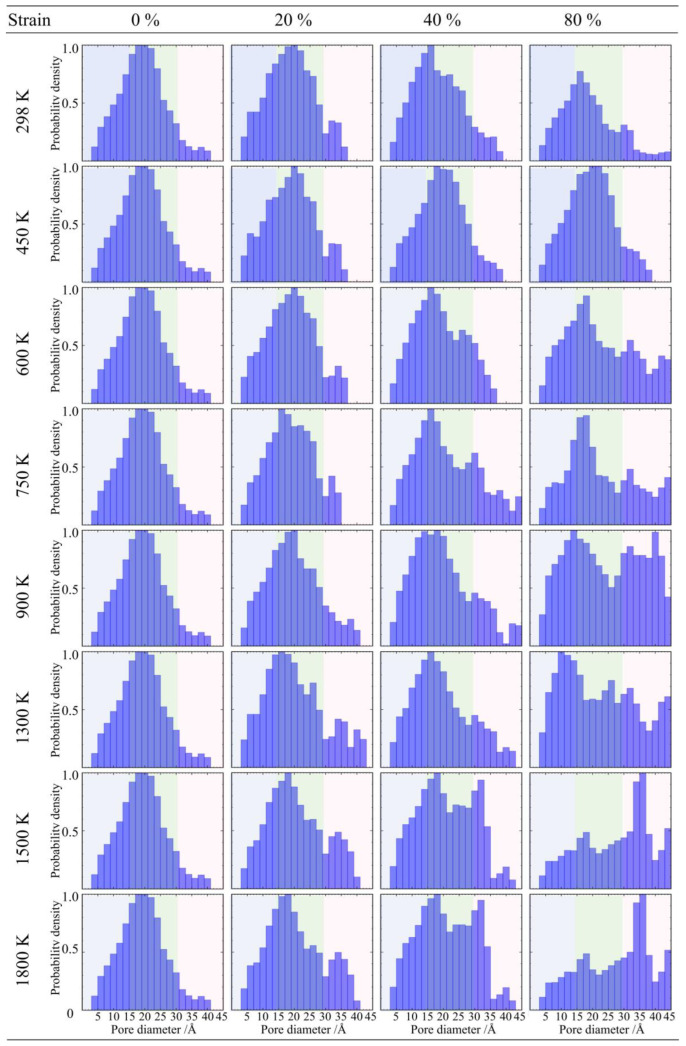
Evolution of pore size distribution for silica aerogel with a density of 0.43 g/cm^3^ under uniaxial tension.

As shown in Figure 17, when the density is 0.43 g/cm^3^, the evolution of pore size distribution exhibits significant temperature dependence. In the low-temperature range of 298 K to 600 K, the material demonstrates good structural stability. Even when the tensile strain reaches 80%, the pore size distribution curve remains basically stable, and the peak positions and intensities of small pores and mesopores do not shift significantly, indicating that the backbone effectively dissipates stress through local rearrangement within this temperature range, maintaining the integrity of the topological structure [44]. However, when the temperature rises to the transition zone of 750 K to 900 K, structural evolution takes a turn. Under large deformation, the density of the large pore region (>3.0 nm) begins to increase, and the tail of the curve lifts, indicating that high temperatures induce microcrack initiation and pore coarsening [45]. This trend peaks in the high-temperature range of 1300 K to 1800 K. In this stage, the density of large-sized crack pores reaches its maximum, and the pore size distribution broadens. This confirms that at high temperatures, atomic thermal vibration accelerates weak bond dissociation, causing the low-density backbone to undergo thermal fracture and pore structure disintegration in the late stages of large deformation.

**Figure 18:**
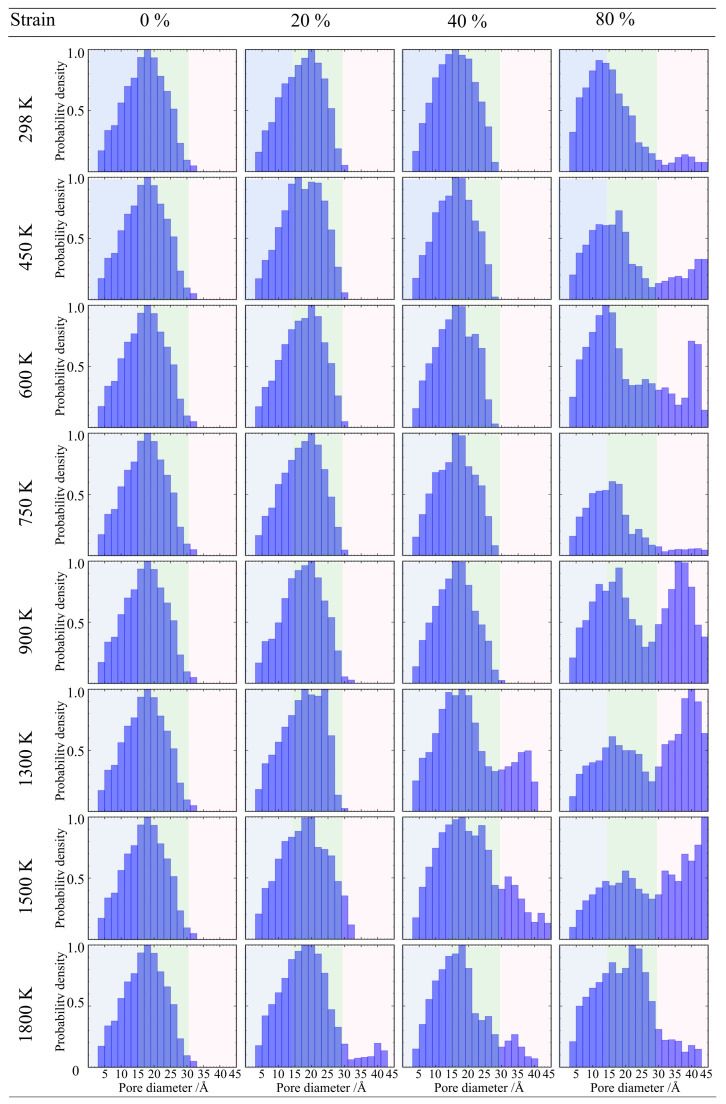
Evolution of pore size distribution for silica aerogel with a density of 0.50 g/cm^3^ under uniaxial tension.

As the density increases to 0.50 g/cm^3^, as shown in Figure 18, the material maintains good topological stability in the low-temperature range of 298 K to 600 K, with no obvious shift in pore size distribution under large deformation. However, entering the medium-to-high temperature range of 750 K to 1300 K, structural damage begins to dominate, and the density of the large pore region shows a stepwise increase with rising temperature, indicating that thermal vibration promotes the coalescence and propagation of microcracks. Nevertheless, when the temperature further rises to 1800 K, the density of the large pore region drops back compared to the peak at 1300 K. This phenomenon indicates that at high temperatures approaching the softening point of silica, the deformation mechanism of the material shifts from brittle fracture to viscous flow; the backbone material blunts crack tips and fills some nascent cavities through viscous flow, thereby offsetting the pore expansion caused by tensile loading to a certain extent [46].

**Figure 19:**
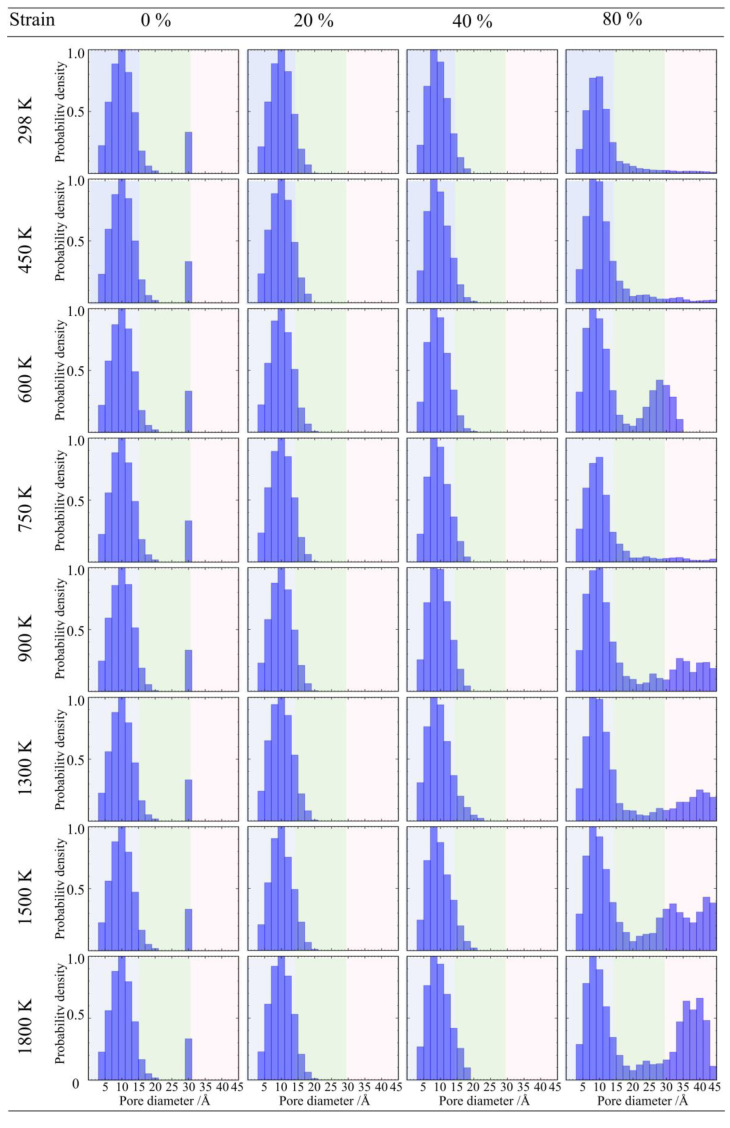
Evolution of pore size distribution for silica aerogel with a density of 0.71 g/cm^3^ under uniaxial tension.

For the high-density sample (0.71 g/cm^3^) (Figure 19), it presents more obvious characteristics of structural damage. In the low-temperature range of 298 K to 600 K, the pore size distribution curve maintains a single-peak morphology. However, as the temperature rises to the 750 K to 1800 K range, the pore size distribution changes, evolving into a bimodal distribution characteristic. In the large pore region (>3.0 nm), an independent new peak appears, and the intensity of this peak increases with rising temperature. This statistical feature corroborates the macroscopic crack opening phenomenon observed in the atomic snapshots earlier, indicating that fracture occurs within the high-density backbone, and crack propagation dominates the rapid increase in pore volume.

#### 2.5.2. Pore Evolution Under Compressive Loading

**Figure 20:**
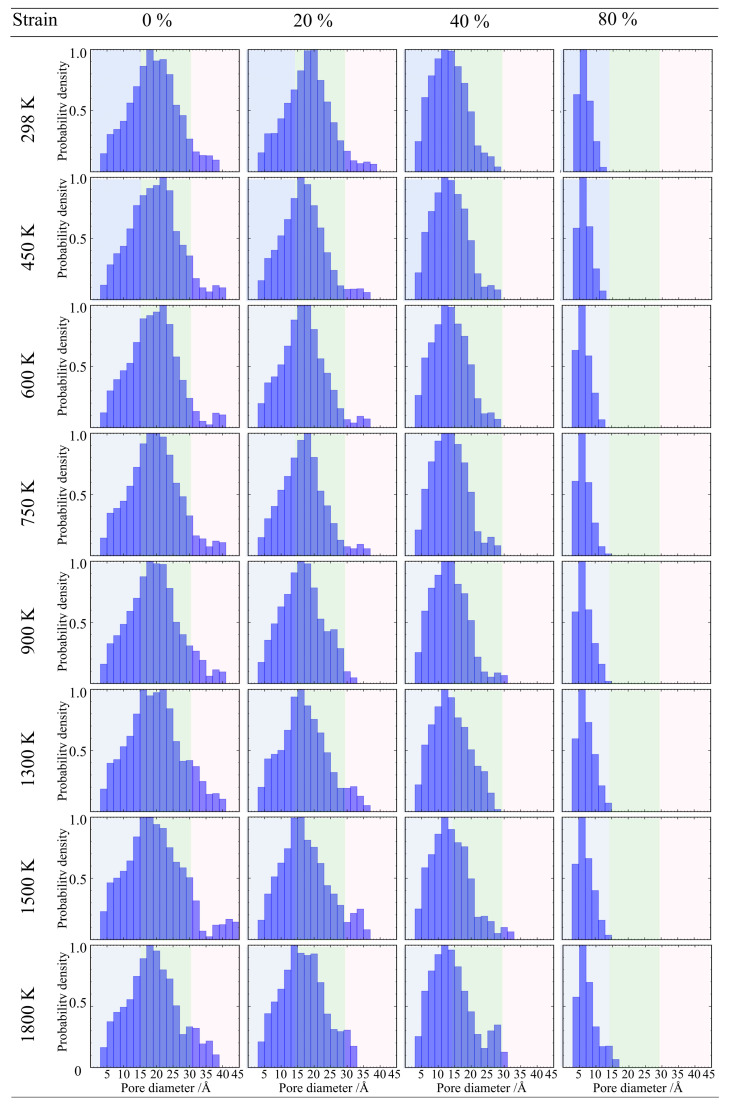
Evolution of pore size distribution for silica aerogel with a density of 0.43 g/cm^3^ under uniaxial compression.

For the low-density silica aerogel (0.43 g/cm^3^), its microscopic pore structure exhibits a characteristic transition from large pore collapse to overall microporosity under compressive loading, as shown in Figure 20. From the start of compression to the yielding stage (strain 20–40%), structural evolution mainly manifests as the closure of large-sized pores (>3 nm) and mesopores, causing the tail of the probability density distribution curve to contract and shift to the left; as the load continues to increase to the densification stage (strain 80%), the free volume inside the backbone is exhausted, and the originally broad pore size distribution eventually converges to a narrow peak located in the 0.5–1 nm interval, indicating that the loose nanoporous network has been compacted into a continuous solid with tight atomic packing, and the atomic diffusion and sintering effects in the high-temperature environment further promote the homogenization of this dense structure [47].

**Figure 21:**
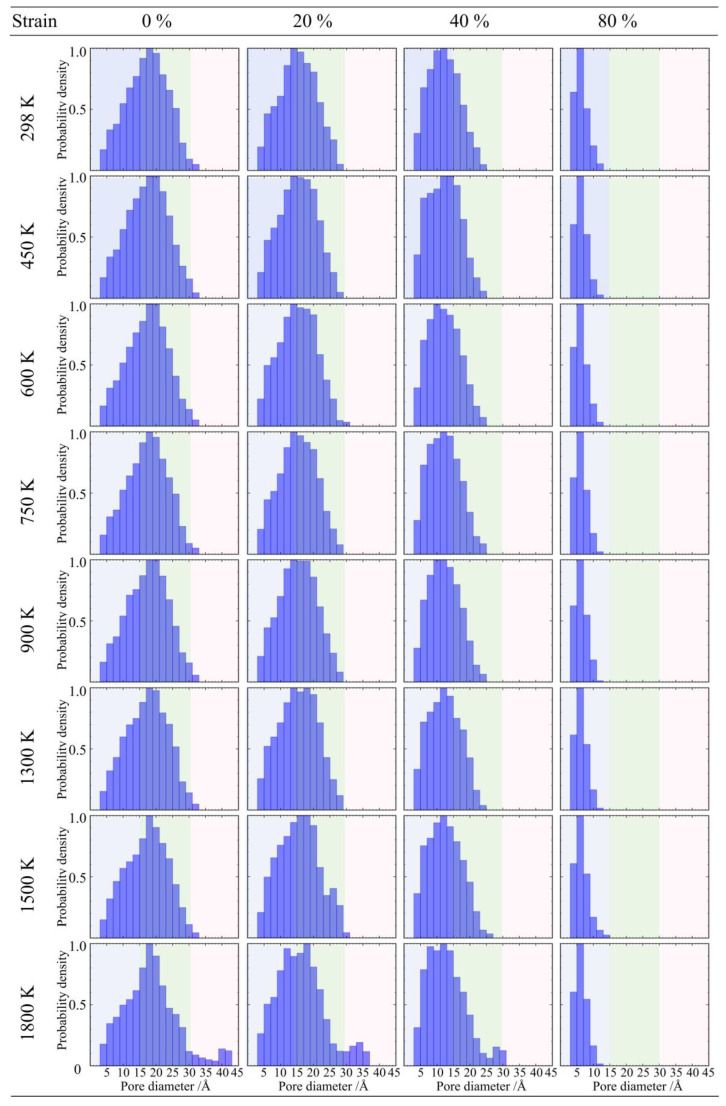
Evolution of pore size distribution for silica aerogel with a density of 0.50 g/cm^3^ under uniaxial compression.

As shown in Figure 21, the medium-density silica aerogel (0.50 g/cm^3^) exhibits pore evolution laws consistent with the low-density sample. Accompanying the increase in compressive strain, the pore size probability density distribution curve of this sample similarly shows a trend of convergence from a broad distribution to a small pore size region. In the limit compression state (strain 80%), regardless of the environmental temperature, its pore structure collapses completely and converges to a narrow peak located in the 0.50–1 nm interval. This indicates that despite different initial densities, the residual microscopic free volumes of aerogel backbones after undergoing complete densification all tend toward the same limit; i.e., retaining only atomic-interstice-scale micropore structures [48].

**Figure 22:**
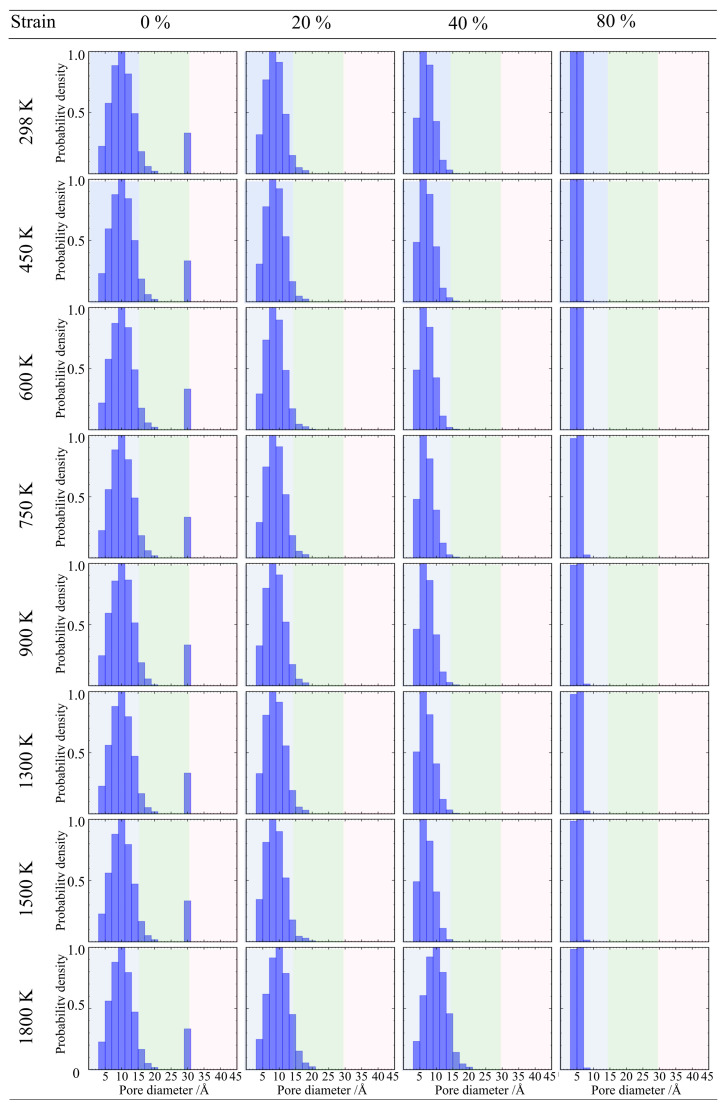
Evolution of pore size distribution for silica aerogel with a density of 0.71 g/cm^3^ under uniaxial compression.

As shown in Figure 22, the high-density silica aerogel (0.71 g/cm^3^) exhibits more pronounced compressive densification characteristics. Unlike medium- and low-density samples, this high-density backbone presents a bimodal distribution characteristic in the initial state, with a small number of discrete large-sized pores (>3 nm) existing in addition to the main mesopore peak; under compressive loading, these large pores with weaker structural stiffness are rapidly crushed and disappear in the early stage of yielding (strain 20%), leading to significant morphological contraction and leftward shifting of the pore size distribution curve. As the compression process deepens to the limit stage (strain 80%), regardless of how the thermal environment changes, the free space inside the material is cleared, and the pore size distribution finally converges to a single and sharp atomic-scale micropore peak (0.55–1 nm), indicating that medium- and high-density samples possess a shorter yielding plateau and enter the densification stage earlier.

## 3. Conclusions

In this work, large-scale molecular dynamics simulations (containing approximately 192,000 atoms) were employed to systematically investigate the effects of density (0.43, 0.50, and 0.71 g/cm^3^) and temperature (298 K to 1800 K) on the mechanical properties and microstructural pore evolution of silica aerogels. Based on the simulation results and micromechanical analysis, the following main conclusions are drawn:

(1) The mechanical properties under tensile loading exhibit a decaying trend with increasing temperature, primarily attributed to the dominance of the thermal softening mechanism. As the temperature rises, intensified atomic thermal vibrations weaken the binding energy of Si-O bonds, making the backbone more prone to thermal fracture, thereby reducing the elastic modulus and fracture strength. Quantitative analysis indicates that even the high-density sample (0.71 g/cm^3^) with good initial connectivity is affected by high temperatures, with its elastic modulus at 1800 K decreasing by approximately 11.1% compared to room temperature
(2) The high-temperature-induced sintering mechanism leads to distinct strengthening characteristics in the compressive elastic modulus, an effect that is more pronounced in high-density samples. When the temperature exceeds 900 K, the backbone fusion and densification caused by sintering outweigh the weakening effect of thermal softening, causing the compressive elastic modulus of the material to rise. Data show that the elastic modulus of the high-density sample (0.71 g/cm^3^) at 1300 K increases by approximately 20% compared to that at 900 K, indicating that high-temperature sintering can effectively enhance the load-bearing capacity of the material.
(3) The non-linear changes in macroscopic mechanical behavior originate from the distinct evolutionary paths of the microscopic pore structure. Pore size distribution analysis reveals that under tensile loading, high temperatures induce pore coarsening and microcrack initiation, evidenced by the emergence of a new peak in the large pore region (>3.0 nm) for the high-density sample, which disrupts backbone continuity and triggers performance degradation. Conversely, under compressive loading, structural evolution is characterized by the rapid collapse and closure of large pores; regardless of the initial density and temperature, the final pore size distribution converges to a narrow atomic-scale micropore interval of 0.5–1.0 nm, indicating that compaction and densification occur within the material.
(4) Density and temperature exert significant coupled regulatory effects on the mechanical properties of aerogels. Density determines the baseline initial mechanical strength, while temperature dictates the specific failure modes. Low-density samples (0.43 g/cm^3^) are severely affected by thermal softening, evidenced by a 46% decrease in compressive elastic modulus from 900 K to 1300 K. In contrast, high-density samples (0.71 g/cm^3^), by virtue of their tighter atomic packing, effectively utilize the sintering mechanism to achieve structural strengthening, showing a 20% increase in compressive elastic modulus within the same temperature range. This finding provides a theoretical basis for designing the microstructure of silica aerogels tailored to specific thermal environments.

While this molecular dynamics study offers theoretical insights into high-temperature strengthening mechanisms at the nanoscale, experimental verification remains crucial for practical engineering applications. Consequently, future work is planned to bridge this gap by fabricating silica aerogel samples with the specific densities investigated in this study and conducting macroscopic thermal safety tests, such as nail penetration or thermal abuse protocols. These follow-up studies aim to quantitatively validate the extent to which the sintering-induced stiffening and pore densification behaviors predicted by the simulations contribute to the structural integrity and thermal isolation of battery modules under real-world thermal runaway conditions.

## 4. Materials and Methods

### 4.1. Model of Porous Silica

MD simulations were performed using the open-source code LAMMPS. The interactions between silicon and oxygen atoms were described by the Tersoff potential [49], which has been proven to accurately reproduce Si-Si and Si-O bonding characteristics and outperforms potentials such as BKS in predicting structural properties [50].

The porous model of silica aerogel was constructed based on the negative pressure principle [34]. First, the amorphous silica matrix was prepared using a melt-quench method: fully dense β-cristobalite crystals were heated to 7000 K for complete melting, followed by rapid quenching to 300 K. Radial distribution function (RDF) analysis showed that the obtained Si-O, Si-Si, and O-O bond lengths were 1.609 3.066, and 2.626 Å, respectively, which agree well with literature data [50], verifying the accuracy of the initial structure. Subsequently, negative pressure was introduced via volume expansion to reduce the matrix density to the target values. In this work, models with three densities, 0.43, 0.50, and 0.71 g/cm^3^, were constructed. Each system contained over 192,000 atoms to eliminate size effects [34]. All generated models underwent high-temperature relaxation and equilibration to form a stable nanoporous network for subsequent mechanical testing.

### 4.2. Molecular Simulation Settings

This study simulated the mechanical behavior of silica aerogels within a temperature range of 298 to 1800 K. Atomic velocities at each temperature were initialized according to the Maxwell–Boltzmann distribution [51]. The system first underwent thermodynamic relaxation for 500 ps under the NPT ensemble. The Nosé–Hoover thermostat and barostat were used to strictly control temperature and pressure, eliminating residual modeling stresses and ensuring the system reached an equilibrium state of energy and volume prior to deformation. The time step for deformation simulation was set to 1.0 fs. A constant strain rate of 10^9^ s^−1^ was applied along the Z-axis, while a zero-pressure condition controlled by NPT was applied to the lateral directions (X and Y axes), allowing for free transverse deformation. System stress was calculated based on the Virial theorem [52], with stress–strain data and atomic trajectories recorded every 10 ps for microstructural evolution characterization. The application of zero-pressure NPT conditions in the transverse directions during deformation allows the simulation box to freely contract or expand in response to axial loading. This setup enables the simulation box dimensions to adjust according to internal stress variations, thereby facilitating the accurate capture of the Poisson effect and preventing the development of non-physical internal lateral stresses. As the system approaches the fracture stage, it ensures that the failure mechanisms and fracture paths remain physically realistic.

While the Tersoff potential is widely employed for capturing the bond breaking and formation processes essential for fracture simulation, its limitations regarding high-temperature thermodynamics and bond rearrangement kinetics warrant explicit discussion. It is documented that this potential tends to overestimate the melting point of silica, typically predicting Tm > 2600 K. Within the specific context of the present study, however, this overestimation serves a functional methodological purpose. Given the extremely high surface-to-volume ratio of silica aerogels, nanostructured backbones in molecular dynamics simulations often exhibit enhanced surface activity and may undergo premature softening or surface pre-melting on short timescales. The application of a potential with a melting point closer to experimental values could result in non-physical early melting or excessive viscous flow at the simulation temperature of 1500 K, which would obscure the core physical phenomena of interest, specifically the solid-state sintering and densification driven by atomic diffusion.

The high thermal stability provided by the Tersoff potential ensures that the simulated structure retains its amorphous solid-state character at 1500 K, operating at a homologous temperature of approximately T/Tm ≈ 0.58. This stability allows for the isolation and observation of structural reorganization and modulus enhancement within the solid phase, providing a physical model for structural evolution that remains undisturbed by liquid-phase collapse. Regarding bond rearrangement, although the potential reproduces the topological evolution of the network under load, the precise energy barriers for bond switching may differ from higher-level benchmark data. Consequently, the high-temperature structural evolutions reported in this work should be interpreted as providing mechanistic insights into the material topological response and relative strengthening trends, rather than delivering precise quantitative predictions of absolute reaction kinetics or thermodynamic transition temperatures.

### 4.3. Calculation of Pore Structure

To accurately characterize the microstructural evolution of silica aerogels during heat treatment and mechanical loading, this work implemented automated pore structure identification based on the three-dimensional grid method [53]. First, atomic coordinates were extracted frame-by-frame from LAMMPS output trajectory files, and atoms crossing periodic boundaries were remapped to eliminate boundary truncation effects. Subsequently, the simulation system was meshed into uniform three-dimensional grids, and atoms were looped to calculate their spatial distribution. Based on the criteria proposed by Yang et al. [36], regions containing a number of atoms exceeding a threshold were defined as backbone grids, while others were marked as pore grids, effectively achieving the separation of the solid silica backbone from the gas-phase pores.

Building upon pore identification, this study further quantified the pore size distribution. The identified atomic configuration data were converted into standard XYZ format files for geometric statistical analysis. Pore size was defined as the diameter of the largest sphere that could be accommodated at a given spatial point without overlapping with surrounding backbone atoms. Finally, based on the calculation results, the probability density function of pore size was constructed to quantitatively reveal the pore collapse, merging, and coarsening behaviors of the aerogel under different thermodynamic conditions.

## Figures and Tables

**Table 1:** Comparison of research scopes between prior studies and the present work.

Category	Typical References	Conditions	Focus	Identified Gap
Experimental Studies	Yang et al. [32], Iswar et al. [33]	298 K or Post-heat treatment	Macroscopic strength, creep, and scaling laws	Limited real-time observation of atomic-scale evolution under extreme heat
MD Simulations (Room Temp)	Patil et al. [34], Lei et al. [35]	298 K	Topological connectivity and mechanical power laws	Neglect of thermal activation and kinetic structural reorganization
MD Simulations (Thermal Focus)	Yang et al. [36]	298–1500 K (Static)	Sintering mechanism and thermal conductivity	Lack of mechanical loading coupling and stress-induced failure modes
Present Study	This Work	298–1800 K (Dynamic Coupling)	Competition between softening and sintering under load	Fill the gap in real-time thermo-mechanical response at extreme temperatures

## Data Availability

Data is provided within the manuscript.
